# *QuickStats:* Percentage* of Employed Adults Who Needed to Work Closer Than 6 Feet from Other Persons All or Most of the Time at Their Main Job,^†^ by Occupation^§^ — National Health Interview Survey, United States, July–December 2020^¶^

**DOI:** 10.15585/mmwr.mm7049a7

**Published:** 2021-12-10

**Figure:**
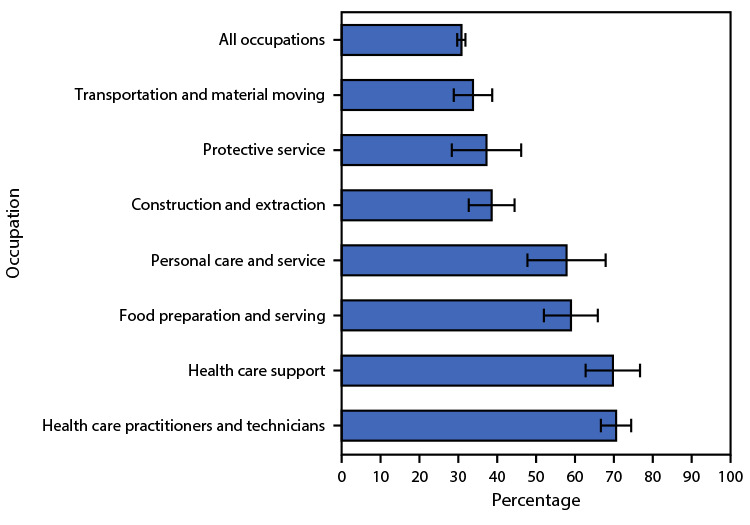
During July–December 2020, 30.7% of all currently employed workers needed to work closer than 6 ft (2 m) from other persons at their job all or most of the time. The four occupations with the highest percentages were health care practitioners and technicians (70.5%), health care support (69.7%), food preparation and serving (58.9%), and personal care and service (57.8%) occupations.

**Source:** National Health Interview Survey, 2020. https://www.cdc.gov/nchs/nhis/2020nhis.htm

For more information on this topic, CDC recommends the following link: https://www.cdc.gov/niosh/emres/2019_ncov_default.html

